# Residual deficits of knee and hip joint coordination and clinical performance after return to sports in athletes with anterior cruciate ligament reconstruction

**DOI:** 10.1186/s43019-024-00213-w

**Published:** 2024-06-17

**Authors:** Komsak Sinsurin, Pongthanayos Kiratisin, Dimas Sondang Irawan, Roongtiwa Vachalathiti, Jim Richards

**Affiliations:** 1 Biomechanics and Sports Research Unit, Faculty of Physical Therapy, Mahidol University, Salaya, Nakhon Pathom, 73170 Thailand; 2 Physical Therapy Department, Faculty of Health Sciences, University of Muhammadiyah Malang, Malang, Indonesia; 3 Musculoskeletal Physical Therapy Research Unit, Faculty of Physical Therapy, Mahidol University, Salaya, Nakhon Pathom, Thailand; 4 Allied Health Research Unit, University of Central Lancashire, Preston, UK

**Keywords:** Anterior cruciate ligament reconstruction, Knee coordination, Limb symmetry index, Recurrent ACL injury, Return-to-sport

## Abstract

**Background:**

Biomechanical changes and neuromuscular adaptations have been suggested as risk factors of secondary injury in individuals after anterior cruciate ligament reconstruction (ACLr). To achieve a better understanding of preventive mechanisms, movement quality is an important factor of consideration. Few studies have explored time-series analysis during landing alongside clinical performance in injured and non-injured individuals. The purpose of the study was to investigate the biomechanical risks of recurrent injury by comparing clinical and jump-landing performance assessments between athletes with ACLr and healthy controls.

**Method:**

This study was observational study. Sixteen athletes with and without ACLr voluntarily participated in clinical and laboratory measurements. Single-leg hop distance, isokinetic tests, landing error score, and limb symmetry index (LSI) were included in clinical report. Lower limb movements were recorded to measure joint biomechanics during multi-directional landings in motion analysis laboratory. Hip-knee angle and angular velocity were explored using discrete time-point analysis, and a two-way mixed analysis of variance (2 × 4, group × jump-landing direction) was used for statistical analysis. Time series and hip-knee coordination analyses were performed using statistical parametric mapping and descriptive techniques.

**Results:**

Significantly lower single-leg hop distance was noted in ACLr group (158.10 cm) compared to control group (178.38 cm). Although the hip and knee moments showed significant differences between four directions (*p* < 0.01), no group effect was observed (*p* > 0.05). Statistical parametric mapping showed significant differences (*p* ≤ 0.05) between groups for hip abduction and coordinate plot of hip and knee joints. Athletes with ACLr demonstrated a higher velocity of hip adduction. Time-series analysis revealed differences in coordination between groups for frontal hip and knee motion.

**Conclusions:**

Athletes with ACLr landed with poor hip adduction control and stiffer knee on the involved side. Multi-directions landing should be considered over the entire time series, which may facilitate improved movement quality and return to sports in athletes with ACLr.

## Introduction

Anterior cruciate ligament (ACL) injury is one of the most common and serious injuries of the lower extremity in sports, especially in jump-landing and cutting sports such as volleyball, basketball, and football [1, 2]. The ACL reconstruction (ACLr) is believed to be the gold standard treatment and has shown a high success rate for return to normal knee function and level of sports activities [3, 4]. After ACLr, 82% of athletes can return to participation in some sports and 44% can return to competitive sports [5]. However, a high rate of secondary ACL injury has been reported, with repeat injury rates of 12 to 17.2% within 5 years after ACLr [6, 7], and with on 7% ipsilateral and 8% contralateral sides [8]. Additionally, individuals with ACLr have been reported to be at 15-times higher risk for injury than their counterparts without ACL injury [9]. The mechanisms of secondary ACL injury have been reported to be due to similar movement patterns as the first injury [10], and biomechanical changes and neuromuscular adaptations have been suggested as the main risk factors [11]. Previous studies have investigated the biomechanics of landing adaptations after ACLr, with the ACLr group showing a stiff leg pattern compared to the non-injured group. Kinetic data have also been reported to show reduced peak hip and knee-extensor joint moments in an ACLr group representing lower work done by the hamstring and quadriceps muscles during energy absorption [12, 13].

Many evaluations such as isokinetic tests and hop tests have been used in the decision-making process when assessing whether athletes who had undergone ACLr can return to sports [14]. These tests evaluate the symmetry between the limbs based on quantitative data. However, there is no gold standard test for determining whether it is appropriate to return to sports. Many criteria from quantitative data have been suggested, including the limb symmetry index (LSI) of hop distance and isokinetic tests, and clinical symptoms [15]. Lack of qualitative assessment may be a limitation when screening for the risk of primary or recurrent ACL injury. The assessment of movement quality has been suggested to determine the readiness of return to sports [16]. To study movement quality, coordination of lower limb joints reflects motor control from the central nervous system [17] and has been studied using angle-angle plots, velocity-angle plots [18, 19], and angular velocity [20]. Changes in movement coordination have been demonstrated in individuals with knee pain by comparing them with healthy individuals [21]. These changes have been associated with joint instability resulting in neuromuscular impairment [22].

To achieve a better understanding of preventive mechanisms, identify the risk of injury, and identify the ability to return to sports, movement coordination is an important factor of consideration [23]. The current study examined the clinical impairment and quality of hip and knee movements during jump-landing task in one single study. The findings would provide additional insights to develop movement strategies for athletes with ACLr after return to sports. Therefore, the primary purpose of the current study was to compare clinical and jump-landing performance assessments between athletes with ACLr and those in the healthy control groups. The secondary purpose was to consider the hip and knee movement coordination during jump landing in the forward, diagonal, and lateral directions between the ACLr and control groups. We hypothesized that athletes with ACLr might show movement risks of secondary injury on jump-landing performance assessments.

## Material and methods

### Participants

This observational study was conducted in a movement analysis laboratory. The research protocol was approved by the Mahidol University Central Institutional Review Board for Human Research (MU-CIRB 2016/051.0704), and all participants provided written informed consent prior to data collection. G*Power (version 3.0.10) was used to estimate the sample-size for the laboratory performance test. Pilot data from the knee extensor moment was used to estimate the sample-size for the laboratory performance tests with a probability level of 0.05 (*p*-value) and a power analysis of 80%, which led to seven athletes per group. It is important to note that an athlete from the 10% drop-out calculation was added to each group.

Sixteen athletes voluntarily participated and were divided into an ACLr group (8 athletes with ACLr) and a control group (8 healthy controls) who regularly performed jump-landing sporting tasks. In the ACLr group, participants had previously undergone ACLr with bone-patellar-bone or hamstring-tendon graft techniques. The athletes in the ACLr group were evaluated for their level of sport using the Tagner activity level scale. All athletes in the ACLr group had returned to competitive sports or participated at the same level of sports as their pre-injured status. Patients who had undergone an ACLr were excluded if they had a total or partial meniscectomy, serious injury of the hip or ankle joint, or history of low back pain 6 months prior to the study by receiving medication or undergoing physical therapy. Participants’ characteristics in the ACLr and control groups were matched for sex, age, sport type, and sport performance level.

### Clinical assessments

Athletes were assessed using clinical performance tests, including a single-leg hop-distance test, isokinetic test, and double-leg jump-landing test. The landing error score system (LESS) was used to assess the risk of knee injury during the double-leg jump-landing test [25]. A score of more than 5 points according to the LESS indicated a high risk of ACL injury during landing [26]. Knee extensor and flexor muscle strength were tested using an isokinetic dynamometer (Biodex System 4; Biodex Medical System Inc., New York, NY, USA).

### Three-dimensional movement analysis

The lower limb kinematics and kinetics during single-leg jump-landing tasks were recorded using a 10 camera Vicon™ Nexus system (Oxford Metrics, Oxford, UK) at 100 Hz and an AMTI force plate (Advanced Mechanical Technology, Watertown, MA, USA) at 1000 Hz. Sixteen reflective markers were attached bilaterally to the anterior superior iliac spine, posterior superior iliac spine, thigh, lateral condyle of the femur, shanks, lateral malleoli, heels, and the head of the second metatarsal bones.

In real sports games and practice, athletes perform landings in multiple directions. Therefore, adding the directional complexity in jump-landing tasks would increase the challenge for assessing lower extremity biomechanics in athletes with ACLr after returning to sports. A 30-cm platform was positioned 70 cm from the force plate center, and participants were asked to jump and land from four directions: forward (0°), 30° diagonal, 60° diagonal, and lateral (90°) directions based on a previously published protocol [27–29]. The ACLr limb and matched-paired side of the control group (dominant or non-dominant) was tested and compared between the ACLr and control groups.

### Data acquisition and statistical analysis

LSI between the sides was calculated and reported based on forward single-leg hop distance, knee flexor strength, and knee extensor strength. Statistical analyses were performed using the SPSS version 17 (IBM, Armonk, NY, USA). Data from the clinical performance tests were compared between the ACLr and control groups using independent *t*-tests and Mann–Whitney *U* tests, depending on the distribution of the data.

The marker and force data were filtered using a fourth-order zero-lag Butterworth digital filter at cut-off frequencies of 8 Hz and 40 Hz, respectively. The cut-off frequencies were determined using the residual analysis technique [30]. A three-dimensional model was constructed using Visual3D version 6 (C-Motion Inc., Germantown, MD, USA). Knee and hip joint kinematics were calculated based on the Cartesian sequence of XYZ, which is equivalent to the joint coordinate system proposed by Grood and Suntay [24]. An average of three successful trials in each jump-landing direction for the tested limb was analyzed. The operational definition of the landing phase was identified from initial contact (IC) to 300 ms after the IC. The hip and knee flexion excursion angles and angular velocities at the IC and peak vertical ground reaction force (vGRF) were extracted. Flexion excursion angles were calculated from the angular displacement from the IC to the peak flexion during the landing phase. At peak vGRF, the net joint moments for the hip and knee joints in the sagittal plane were extracted.

Kinematic and kinetic data were found to be suitable for parametric testing using Shapiro–Wilk normality tests. Mixed model analysis of variance tests (2 × 4, group × jump-landing direction) were used to analyze the effect of group and jump-landing direction. In addition, post-hoc pairwise comparisons with the Bonferroni correction were performed to compare the landing directions. Statistical significance was set at an alpha level of 0.05.

It has been suggested that discrete point analysis may not be able to determine some of the important differences in joint kinematics and kinetics [24]. Therefore, this study also explored the use of statistical parametric mapping (SPM) of the kinematic time-series data. To compare joint angles and velocities of the hip and knee throughout the landing phase, the complete angle and velocity time-series were analyzed to compare between the athletes with ACLr and healthy controls using SPM (two-sample *t*-test) [31, 32]. For movement quality assessment, plots of joint coordination were produced, including the hip-knee angle and angular velocity angle of the hip and knee joints in the sagittal and frontal planes.

## Results

The athletes’ characteristics are shown in Table 1. Each group comprised six males and two females who regularly played football (*n* = 4), basketball (*n* = 2), rugby (*n* = 1), and volleyball (*n* = 1). No significant differences in age, weight, height, and body mass index were observed between the participants in ACLr and control groups.

**Table 1:** Characteristics of participants

Characteristics	ACLr group (*n* = 8)				Control group (*n* = 8)				*P*-value
	Mean	SD	95% CI		Mean	SD	95% CI		
			Lower	Upper			Lower	Upper	
Age (years)	22.3	2.3	20.4	24.1	20.8	2.0	19.1	22.4	0.18
Weight (kg)	70.4	6.0	65.4	75.3	72.2	8.6	65.0	79.4	0.63
Height (cm)	173.4	6.1	168.3	178.5	173.8	5.8	169.0	178.7	0.89
BMI (kg/m^2^)	23.4	0.8	22.7	24.0	23.8	1.9	22.3	25.4	0.53
Time after surgery (month)	17.5	9.9	9.2	25.8					
Sex (male/female)	6/2				6/2				

*ACLr* anterior cruciate ligament reconstruction, *BMI* body mass index, *CI* confidence interval, *SD* standard deviation

### Clinical assessments

The findings of the clinical parameters are reported in Table 2, and significant differences were observed in single-hop distance, H:Q ratio, and LSI for knee extension strength.

**Table 2:** Comparison of clinical parameters between the ACLr and control groups

Parameters	ACLr group (*n* = 8)					Control group (*n* = 8)					*P*-value
	Mean	SD	Median	95% CI		Mean	SD	Median	95% CI		
				Lower	Upper				Lower	Upper	
LESS score	2.42	1.31	2.33	1.32	3.51	2.46	1.30	2.50	1.37	3.54	0.95 ^**a**^
Single-leg hop distance (cm)	158.10	16.21	156.17	144.55	171.66	178.38	7.82	178.5	171.84	184.91	**0.02** ^**b**^
Peak torque of knee extension (% body mass)	194.75	47.45	193.50	155.07	234.42	224.58	24.61	233.20	204.00	245.16	0.14 ^**a**^
Peak torque of knee flexion (% body mass)	115.17	27.83	115.30	91.90	138.43	107.27	22.23	112.15	88.68	125.86	0.54 ^**a**^
Knee *H*:*Q* ratio (%)	60.19	8.94	59.05	52.72	67.66	47.44	9.66	47.10	39.36	55.51	**0.02** ^**a**^
LSI of knee extension (%)	90.38	18.63	92.31	74.81	105.95	104.48	6.72	104.125	98.87	110.10	0.06 ^**a**^
LSI of knee flexion (%)	105.45	8.48	104.38	98.36	112.53	104.99	18.53	111.13	89.50	120.49	0.46 ^**b**^
LSI of single leg hop distance (%)	99.58	6.17	102.27	94.42	104.73	96.38	7.26	96.50	90.31	102.45	0.35 ^**b**^

Bold values indicate statistically significant differences (*p* < 0.05) between ACLr and control groups

*ACLr* anterior cruciate ligament reconstruction, *CI* confidence interval, *H:Q* hamstring:quadriceps, *LESS* Landing Error Score System, *LSI* limb symmetry index, *SD* standard deviation

^a^Statistical testing with independent *t* test

^b^Statistical testing with Mann–Whitney *U* test

### Three-dimensional movement analysis

**Fig. 1:**
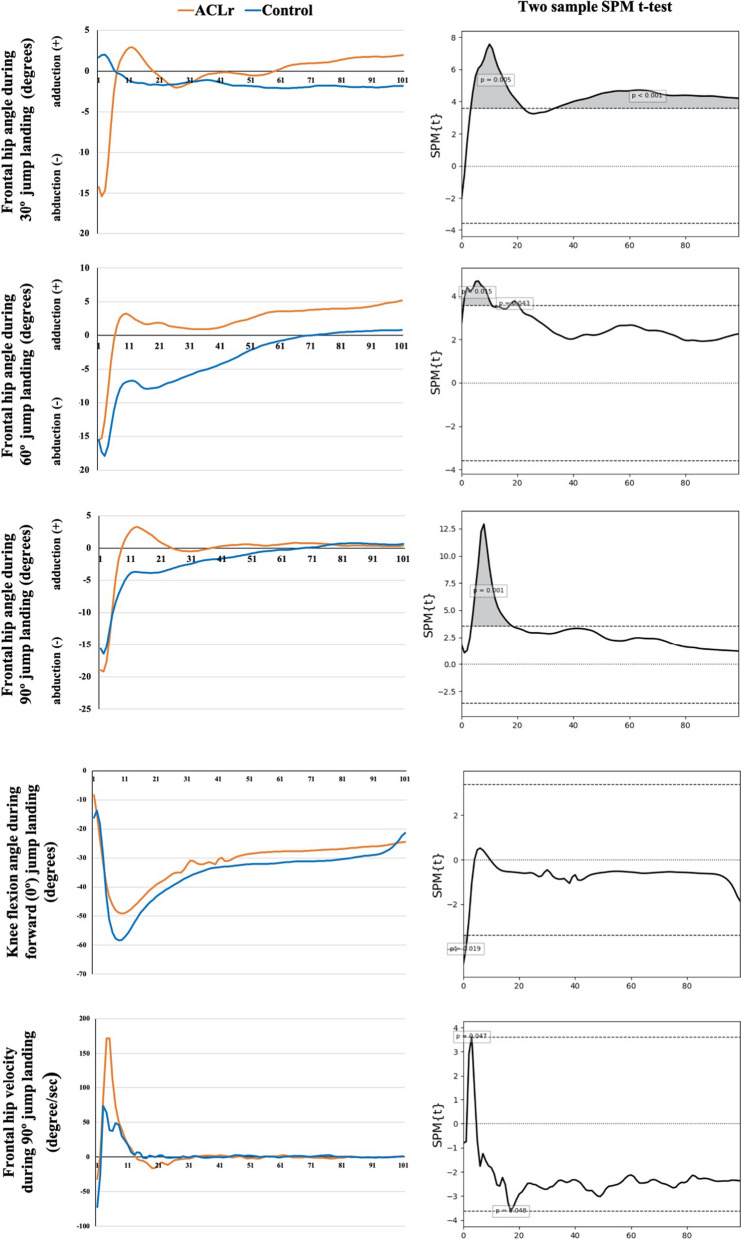
Two-sample SPM *t*-test results between the ACLr (*n* = 8) and control (*n* = 8) groups. *ACLr* anterior cruciate ligament reconstruction, *SPM* statistical parametric mapping

**Fig. 2:**
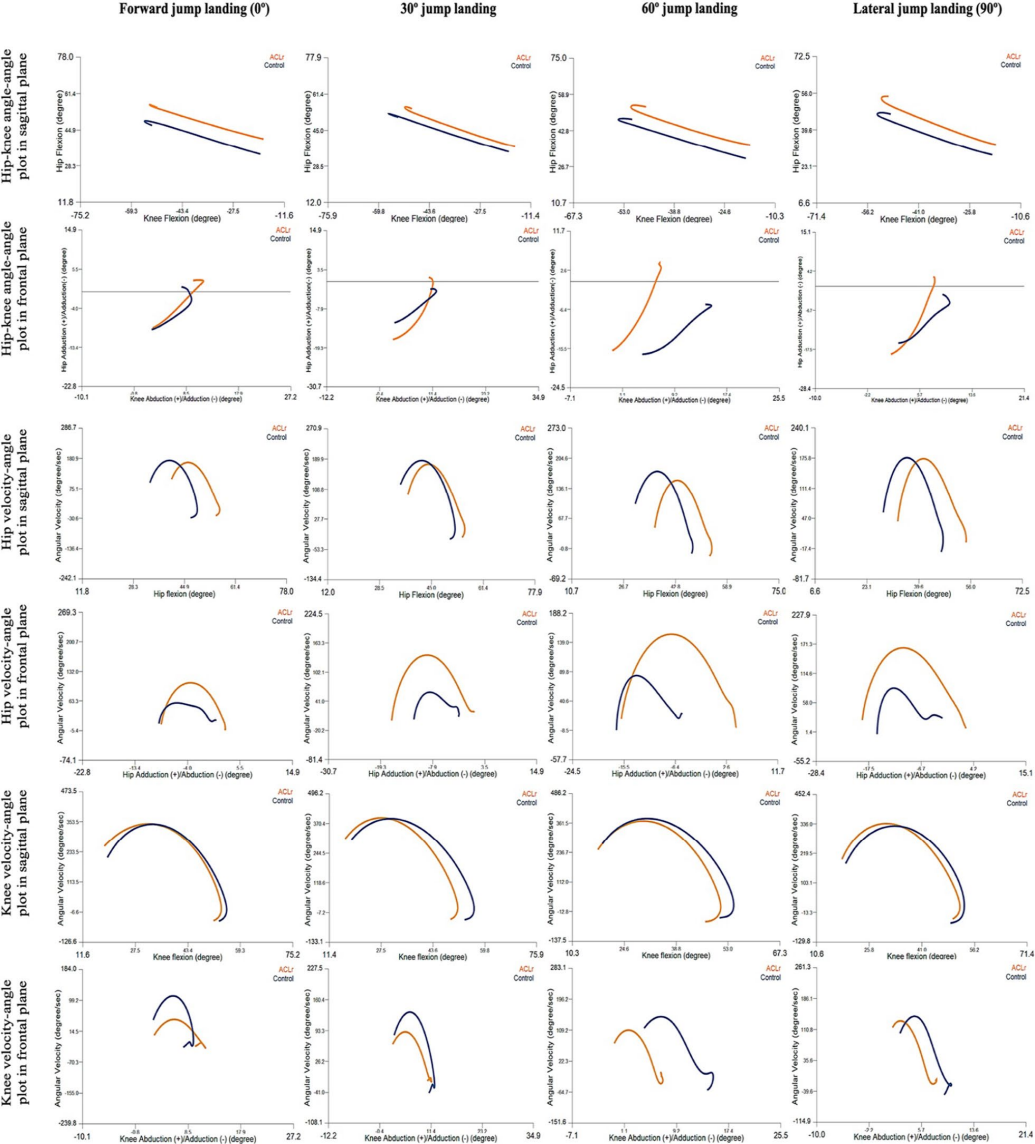
Hip-knee angle and velocity plots in sagittal and frontal planes between the ACLr (*n* = 8) and control (*n* = 8) groups in different directions of jump landing. *ACLr* anterior cruciate ligament reconstruction

The joint kinematics and kinetics of the hip and knee for the jump-landing tests are presented in Table 3. No significant interaction effects were observed between the group and jump-landing directions. Significant main effects were observed for the direction of hip angular velocity at IC, knee angular velocity at IC and at peak vGRF, and net joint moments of lower limb joints at peak vGRF; however, no main effects were observed between groups. However, the SPM time-series analysis showed significant differences between the ACLr and control groups during 30°-, 60°-, and 90°-jump landing for frontal hip motion (Fig. 1), with the ACLr group showing higher velocity of hip adduction motion in the early phase and greater hip adduction during landing. In addition, coordinate plots for the hip and knee showed differences in angle and angular velocity, especially in the frontal plane between the ACLr and control groups, with ACLr athletes landing with a greater frontal hip motion and less movement into knee flexion (Fig. 2).

**Table 3:** Comparison of sagittal joint kinetics and kinematics in multi-directions jump landing between the ACLr and control groups

Athlete	Direction		Hip excursion (degrees)	Hip angular velocity at IC (degrees/sec)	Hip angular velocity at peak vGRF (degrees/sec)	Knee excursion (degrees)	Knee angular velocity at IC (degrees/sec)	Knee angular velocity at peak vGRF (degrees/sec)	Hip extensor moment at peak vGRF (Nm/kg)	Knee extensor moment at peak vGRF (Nm/kg)	Ankle plantar flexor moment at peak vGRF (Nm/kg)
ACL group (*n* = 8)	0° (Forward)	Mean	17.5	109.1	179.3	39.7	278.3	367.2	3.02 ^c^	0.53	2.44
		SD	4.3	34.4	25.1	10.0	58.4	49.0	0.44	0.24	0.25
		95% CI	11.2–23.8	44.4–173.8	120.6–237.9	30.3–49.0	201.9–354.6	300.6–433.8	2.08–3.97	0.01–1.04	1.91–2.98
	30° diagonal	Mean	18.8	103.7 b	178.3	35.6	309.9 ^c^	375.9^c^	3.20 ^c^	0.51	2.29^b,c^
		SD	3.6	44.4	28.4	9.5	70.6	53.4	0.35	0.22	0.27
		95% CI	12.4–25.2	60.5–146.9	136.4–220.1	27.0–44.2	252.7–367.0	328.9–423.0	2.46–3.95	0.05–0.97	1.71–2.87
	60° diagonal	Mean	19.1	52.9^a^	160.7	36.4	255.2	376.1	3.23^c^	0.53 ^c^	2.73
		SD	4.2	26.3	26.5	7.4	67.5	40.9	0.40	0.28	0.20
		95% CI	12.8–25.4	13.2–92.6	121.3–200.0	29.7–43.0	202.1–308.3	328.1–424.2	2.38–4.08	−0.08–1.14	2.30–3.15
	90° (Lateral)	Mean	22.8	40.3	177.2	35.6	196.0^a^	343.4^a^	4.11	0.10	2.93
		SD	7.3	22.2	34.2	9.6	31.6	53.9	0.35	0.27	0.16
		95% CI	15.3–30.2	9.0–71.6	140.8–213.6	27.7–43.4	168.8–223.1	302.7–384.2	3.37–4.86	−0.48–0.68	2.6–3.27
Control group (*n* = 8)	0° (Forward)	Mean	19.1	96.5	193.2	41.5	210.9	355.4	2.24^a,b,c^	1.23^a,b,c^	2.36^a,b,c^
		SD	7.5	81.8	76.4	8.0	86.9	77.1	0.44	2.40	0.25
		95% CI	12.8–25.4	31.8–161.2	134.5–251.8	32.2–50.9	134.5287.2	288.8–422.0	1.3–3.19	0.72–1.75	1.83–2.89
	30° diagonal	Mean	19.1	122.5	190.3	39.9	304.0^c^	393.0^c^	2.96	1.05	2.31
		SD	8.1	39.2	49.9	7.1	34.0	36.3	0.35	0.22	0.27
		95% CI	12.6–25.5	79.3–165.8	148.4–232.2	31.3–48.5	246.8–361.1	345.9–440.1	2.21–3.70	0.59–1.52	1.73–2.89
	60° diagonal	Mean	19.8	101.3	183.3	37.9	274.5 ^c^	368.8	2.95	0.88	2.53
		SD	7..57	47.6	47.0	5.4	27.2	51.7	0.40	0.28	0.20
		95% CI	13.5–26.2	61.7–141.0	144.0–222.7	31.2–44.6	221.4–327.5	320.7–416.8	2.1–3.8	0.27–1.49	2.10–2.96
	90° (Lateral)	Mean	20.4	59.3	184.9	35.8	178.7^a,b^	331.1^a^	3.45	0.42	3.05
		SD	7.1	36.7	36.5	4.8	19.6	14.6	0.35	0.27	0.16
		95% CI	13.0–27.9	61.7–141.0	148.5–221.4	28.0–43.7	151.6–205.9	290.4–416.8	2.71–4.19	− 0.16–1	2.72–3.39
Main effect ANOVA (*p*-value)	Group		0.99	0.39	0.597	0.255	0.491	0.897	0.302	0.129	0.887
	Direction		0.43	**0.03**	0.335	0.217	**0.01**	**0.026**	**0.001**	**0.005**	**0.004**
	Interaction		0.295	0.342	0.719	0.754	0.215	0.609	0.518	0.580	0.767

Bold values indicate statistically significant differences (*p* < 0.05) of direction effect

*ACLr* anterior cruciate ligament reconstruction, *ANOVA* analysis of variance, *CI* confidence interval, *IC *initial contact, *SD* standard deviation, *vGRF* vertical ground reaction force

^a^Statistically significant difference compared with the 30° diagonal direction (< 0.05)

^b^Statistically significant difference compared with the 60° diagonal direction (< 0.05)

^c^Statistically significant difference compared with lateral direction (< 0.05)

## Discussion

The findings of the current study showed that significantly lower single-leg hop distance was noted in ACLr group. Although the hip and knee moments showed significant differences between four directions, no group effect was observed. Statistical parametric mapping showed significant differences between groups for hip abduction and coordinate plot of hip and knee joints. Athletes with ACLr demonstrated a higher velocity of hip adduction. Time-series analysis revealed differences in coordination between groups for frontal hip and knee motion.

### Clinical assessments

The findings from the clinical assessment of performance showed significant differences between ACLr and control groups in hop distance (*p* = 0.02) and *H*:*Q* ratio (*p* = 0.02) (Table 2). LSI of isokinetic and hop-distance tests of > 90% have been suggested as recommended criteria for return to sport [15, 33] and poor outcomes in athletes with ACLr have been associated with low LSI scores for quadriceps strength and forward-hop distance [34, 35]. All athletes who had undergone ACLr and were involved in the current study met the objective criteria for returning to sports, except for knee extension strength (LSI < 90%). However, when compared to the control group, the athletes with ACLr showed less single-leg hop distance and peak knee-extension torque and knee-extension strength LSI. These findings indicate that although the LSI of strength and hop performance are acceptable, there is less strength and hop performance of the reconstructed limb in athletes with ACLr. Additionally, the knee injury risk was assessed using the double-leg jump-landing LESS score, which was less than 5 for both the ACLr and control groups, indicating they were at low risk for ACL injury based on the criteria previously published by Padua et al. [26]. This may indicate that the LESS may have some limitations in detecting risky movements, as most non-contact ACL injuries have been reported during single-leg landings [36]. Previous work has demonstrated that lateral direction of single-leg jump landing has a higher risk of knee injury than diagonal and forward directions [27–29].

### Laboratory measurements

The discrete-point analysis showed significant differences (*p* ≤ 0.05) among jump directions for the knee angular velocity at peak vGRF and both hip and knee joint angular velocities at IC, with lower angular velocities observed during lateral jump landing. However, no significant differences (*p* > 0.05) were observed between the ACLr and control groups. This agrees with the findings of Sinsurin et al. [29], in which a trend towards a decrease in knee angular velocity at IC between forward and lateral direction landings was reported. Lateral jump landings should be performed carefully because of the higher risk of knee injury compared with the forward and diagonal directions [27, 28]. When comparing the ACLr and control groups using time-series analysis of joint angles and angular velocities during landing, significant differences were observed in the frontal plane hip movement during 30°-, 60°-, and 90°-jump landings, which indicates that the discrete-point analysis was unable to identify the periods of differences during these movements. This is supported by the comments by Pataky et al. [32], who proposed the techniques to explore whole time series such as SPM, which may be able to detect previously unreported differences in biomechanical datasets.

The response of the lower joint moment is interesting in observing the loading distribution in athletes with ACLr and that in healthy controls, especially at peak vGRF. When considering the magnitude of the vGRF, it has been reported that higher values may be associated with a higher risk of lower extremity injury [16]. The current study found that, at peak vGRF, the jump direction significantly influenced the sagittal joint moments at the ankle, knee, and hip joints, with an increasing trend of the hip extensor and ankle plantarflexor moments with a decrease in knee extensor moment during landing in the forward, 30°-diagonal, 60°-diagonal, and lateral directions, respectively. Small differences in joint moments were observed between the ACLr and control groups, most notably the knee extensor moment; however, no significant main effects or interaction effects were noted.

**Fig. 3:**
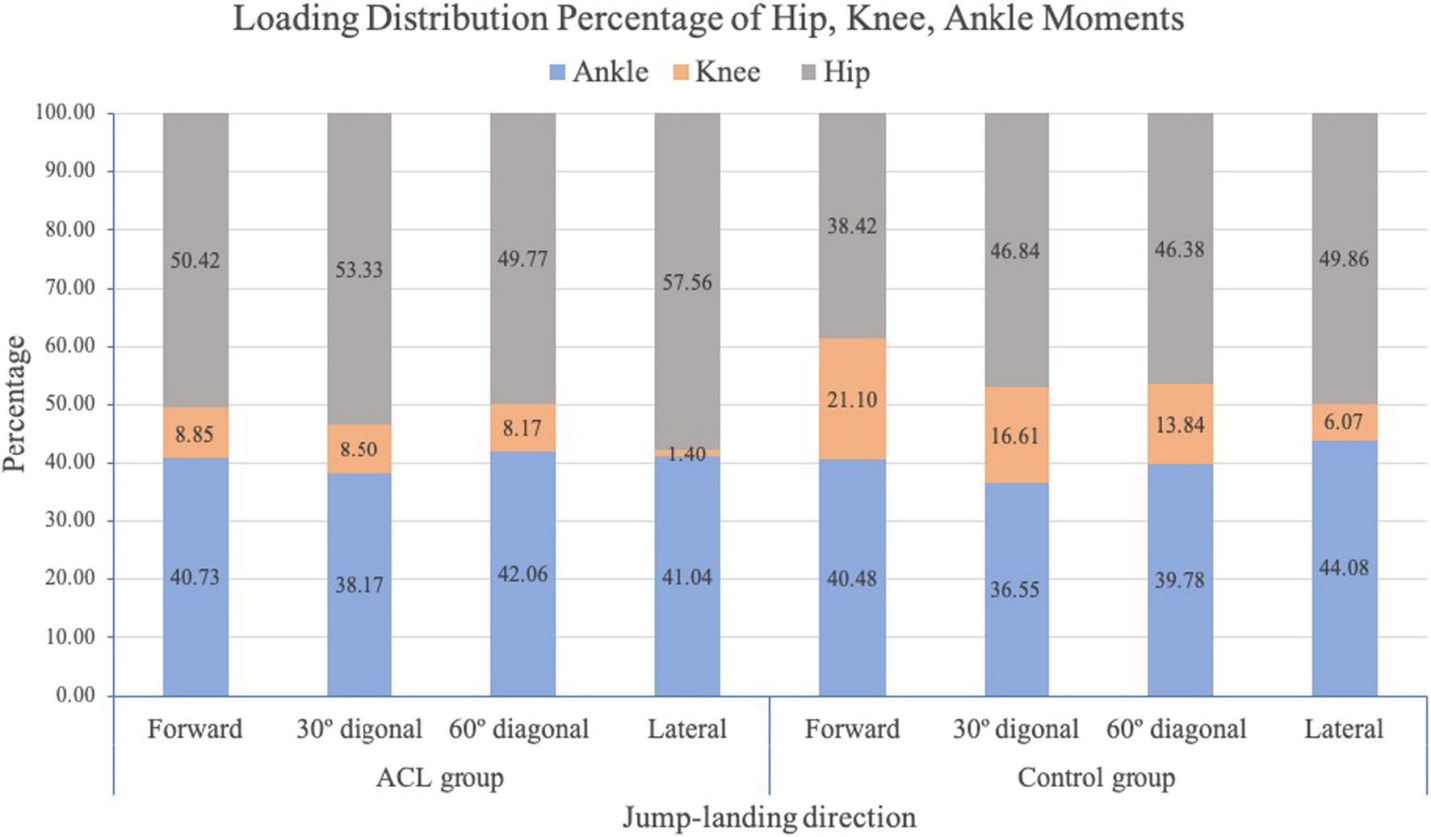
Loading distribution percentage of hip, knee, and ankle moments at peak ground reaction force during landing in different directions

A further analysis explored the percentage of joint loading distribution of the ankle, knee, and hip (Fig. 3). This highlighted that athletes in the ACLr group seemed to prefer using a hip-dominant strategy, while those in the control group seemed to use a hip-and-ankle strategy when responding to the different directions of jump landing. The different movement adaptations of decreasing knee loading between athletes with ACLr and those in the control group might indicate an impairment of loading distribution and control of the lower limb. Further exploration of hip-, knee-, and ankle-loading control is an interesting area for further studies to explore joint kinetic responses in more complex movements.

### Movement quality analysis

Improved joint stability could be the result of improved joint coordination [37], which represents motor control of the higher function of the brain [17]. Previous work comparing diagonal and forward directions of jump landing noted poorer knee coordination during landing in the lateral direction in volleyball athletes [29]. The current study performed descriptive analysis on hip-knee angle-angle plots for the sagittal plane, and the athletes with ACLr generally landed with higher hip flexion than those in the control group, while knee flexion was similar in both groups.

The coordination of hip adduction-knee abduction showed some small differences in pattern between the ACLr and control groups, with the control group showing a consistent response, while the ACLr group showed some differences between jump-landing directions. An increased rate of hip adduction during landing was observed in the ACL group, especially in the diagonal and lateral directions. To prevent the risk of lower extremity injury during landing, eccentric muscle control should be performed effectively to decelerate lower limb movement without collapse. A similar pattern of hip velocity angle between ACLr and control groups was observed in different directions (Fig. 2), which indicates that there are no differences in eccentric control of hip flexion in athletes with and without ACLr. Nevertheless, greater angle of hip flexion was observed in the ACLr group during landing.

Higher magnitudes of velocity were seen in the frontal plane and differences were seen in the hip adduction range during landing in all directions in the ACLr group. This demonstrates that athletes in the ACLr group landed with poor control of hip adduction, which could contribute to the risk of recurrent ACL injury. For athletes who had undergone ACLr, a stiff pattern of knee motion during landing was observed (Fig. 2). This may imply responsiveness during functional demanding tasks through a reduction in the variability of knee movement, which may contribute to the risk of injury [38]. Previous work has reported more rigid movement patterns in athletes with ACL deficiency, which is attributed to movement adaptations [39]. In the sagittal movement, patterns of knee velocity-flexion angle were similar between the ACLr and control groups. However, a greater range of knee flexion was observed in the control group, with a higher magnitude of velocity and range of knee abduction during landing. This indicates less knee control when responding to the different directions of jump landing. This is in contrast to the findings of Pollard et al., in which high variability of two-joint motion during side cutting in athletes with ACLr was reported and was attributed to a lack of feedback from proprioceptive impairment [40].

Our study findings support the conclusions of a previous work by Wiggins et al. [41], who reviewed studies of ACLr and return-to-sport between 1966 and 2015 [41]. They concluded that, after returning to sports, young athletes with ACLr have 30–40 times higher ACL injury risk than uninjured adolescents. In the current study, the angle-angle and velocity-angle plots of the hip and knee joints indicated that, compared to the control group, athletes with ACLr used a greater hip flexion strategy, with the knee flexion showing a trend toward a decrease in eccentric control. This strategy may be a preferred movement in athletes with ACLr who have returned to sports. However, a greater variability of frontal hip motion and velocity was noted. Neuromuscular impairment of the hip abductors might be the reason for poor hip control in the frontal plane. Athletes with ACLr have been shown to have altered brain activity even while performing a simple motion of knee flexion and extension during testing with functional magnetic resonance imaging [42]. Compared to healthy athletes, the contralateral motor cortex, lingual gyrus, and ipsilateral secondary somatosensory area showed increased activity, while the ipsilateral motor cortex and cerebellum activity decreased. We hypothesized that higher brain function in athletes with ACLr might express an impairment of neurocognitive function during jump landing, especially in the frontal movement control of the lower extremity. We suggest that, in screening and evaluation for ACL rehabilitation and return-to-sport assessment guidelines, impaired movement control in the frontal plane should be addressed and corrected to reduce the potential for a recurrent ACL injury.

The findings of this study should be applied to clinical settings carefully, as studies in the laboratory setting do not represent real sports activity, which is more complex, unpredictable, and includes sub-conscious movements. Other limitations include the variety of ACL graft types and lack of control of the rehabilitation protocols before testing. In the future, to better understand, a prospective study should be conducted in athletes with ACL injuries, encompassing time frames from pre- and post-reconstruction, through rehabilitation, return-to-sport consideration, and performance after returning to competition.

## Conclusion

After returning to sports, the discrete parameters of joint kinematics and kinetics in the current study were not different between athletes with ACLr and those in the healthy control group. However, time-series analysis using SPM analysis was able to observe the significant difference of hip adduction motion between the ACLr and control groups in the 30°-, 60°-, and 90°-jump-landing tasks. Qualitative analysis of hip and knee coordination demonstrated differences between ACLr and healthy athletes in the frontal plane control of the hip and knee joints, with athletes with ACLr landing with poorer hip adduction control and a stiff-pattern knee. These findings provide additional insights into the movement strategies in individuals after ACL reconstruction, and the effect of different directions of jump landing should be considered further in future return-to-sport criteria.

## Data Availability

All data from this study is available upon reasonable request to the corresponding author.
